# Genetic Variants in PCSK1 Gene Are Associated with the Risk of Coronary Artery Disease in Type 2 Diabetes in a Chinese Han Population: A Case Control Study

**DOI:** 10.1371/journal.pone.0087168

**Published:** 2014-01-28

**Authors:** Xiaowei Wei, Xiaowei Ma, Ran Lu, Ge Bai, Jianwei Zhang, Ruifen Deng, Nan Gu, Nan Feng, Xiaohui Guo

**Affiliations:** Department of Endocrinology, Peking University First Hospital, Beijing, China; Children's National Medical Center, Washington, United States of America

## Abstract

**Background:**

Insulin and glucagon-like peptide 1 (GLP-1), converted by proprotein convertase 1 (PC1/3) from proinsulin and proglucagon, are associated with type 2 diabetes (T2DM) and coronary artery disease (CAD). The aim of this study is to investigate the association of PCSK1 gene, which encodes PC1/3, with the risk of CAD in Chinese patients with T2DM.

**Methods:**

We selected and genotyped 5 haplotype-tagging single nucleotide polymorphisms (SNPs) at PCSK1 gene (across 39873bp locus) in a case-control study of Chinese Han population involving 425 diabetic patients (62.1% male, mean age 63.2 years) with CAD as positive cases and 258 diabetic patients (44.2% male, mean age 62.0 years) without CAD as controls.

**Results:**

The allele frequencies at rs3811951 were significantly different between cases and controls (30.7% vs. 37.2%), with the allele G associated with decreased risk for CAD (OR = 0.75, 95% CI = 0.59–0.94, p = 0.013). In recessive inheritance mode, the carriers of GG had a lower risk (OR = 0.50, 95%CI = 0.31–0.82, p = 0.005), even after adjusted for gender, age, BMI and smoking (OR = 0.43, 95%CI = 0.24–0.77, p = 0.004). The carriers of the minor allele A at rs156019 had a higher risk (OR = 1.66, 95%CI = 1.10–2.50, p = 0.016 after adjustment) in dominant inheritance mode. The SNP rs6234 was also significantly associated with CAD risk in women, with the carriers of the minor allele G at rs6234 associated with a reduced CAD risk in recessive inheritance mode (OR = 0.42, 95% CI = 0.18–0.95, p = 0.036 after adjustment).

**Conclusions:**

Our results found that common genetic variants in PCSK1 were associated with CAD in Chinese patients with T2DM.

## Introduction

Coronary artery disease (CAD), characterized by the formation of atheromatous plaques, is one of the major complications and accounts for the majority of deaths of type 2 diabetes (T2DM). It has been demonstrated that patients with T2DM had a higher risk of CAD compared to those without T2DM [1]. Meanwhile, the T2DM patients with CAD had shorter life expectancy by 10 years [2]. Atherosclerosis progresses more rapidly in the individuals with T2DM [3]. So, in order to find out the common genetic pathogenesis of CAD and T2DM and reduce the mortality of the two killers, it is clinically critical to screen out the genetic variants at a high risk of CAD in T2DM and further take individualized and positive measures in primary prevention as early as possible.

While relative or absolute lack of insulin is the characteristics of T2DM, insulin is also involved in the development of atherosclerosis. A number of studies showed that insulin exerts protective effects against atherosclerosis through attenuating lipid metabolism, macrophage foam cell formation and oxidative stress [4]. Glucagon like peptide 1 (GLP-1), as another hormone that is developed into an anti-diabetic drug, can inhibit the formation of atheromatous plaques by its regulation of endothelium, vascular smooth muscle cells, monocytes and macrophages [5].

The prohormone convertase 1/3 (PC 1/3), encoded by the PCSK1 gene, functions to convert proinsulin into insulin and proglucagon into GLP-1. Dysfunction of PC 1/3 may induce abnormal release of insulin and GLP-1, thus lead to susceptibility to atherosclerosis. So we hypothesized that genetic variants in PCSK1 might affect the CAD risk through insulin or GLP-1 in the population with T2DM. To confirm the hypothesis, we investigated the CAD-positive and CAD-negative patients with T2DM in Chinese Han population.

## Materials and Methods

### Ethics statement

The study protocol and informed consent procedures were approved by the research ethics committees of Peking University First Hospital. In agreement with the Helsinki Declaration, all subjects had written informed consents to participate in this study.

### Subjects

Diabetes mellitus was diagnosed according to the criteria of the World Health Organization 1999 [6]. Of the total 683 unrelated Chinese Han subjects with T2DM, 425 individuals were CAD-positive and 258 CAD-negative. The CAD-positive cases, defined as those who had a stenosis ≥50% in at least one of major coronary arteries or their main branches on cardiac catheterization at Peking University First Hospital. The CAD-negative controls were confirmed coronary stenosis of any main coronary arteries and main branches were <50% on cardiac catheterization or high specific spiral computer tomography (CT) scan of coronary arteries in Peking University First Hospital. Other demographic data and known cardiovascular risk factors, including gender, age, body mass index (BMI), fasting plasma glucose (FPG), the histories of dyslipidemia, hypertension (blood pressure ≥140/90 mmHg or receiving any antihypertensive therapies) and smoking history (‘ever’ or ‘never’, ‘ever’ defined as having smoked more than 1 cigarette per day for more than 1 year) were collected for all the subjects in their medical records.

### Single nucleotide polymorphism genotyping

Genomic DNA was extracted from peripheral blood leukocytes by salting out procedure (Whole Blood DNA Extraction Kit of bioteke).

PCSK1 in chromosome 5q15–q21 contains 14 exons. Of the total 15 haplotype-tagging SNPs at PCSK1 locus from CHB data in HapMap Phase II database (http://www.hapmap.org) (R#27, r^2^<0.8, MAF≥0.05), 5 SNPs were selected, including rs6230 (T>C), rs6233 (T>C), rs6234 (C>G), rs156019 (T>A) and rs3811951 (A>G), which cover 39873bp of the total 42833bp of PCSK1. Besides coverage, SNPs that are located in exons or found associated with human diseases in previous studies are preferred. The 5 SNPs were genotyped by polymerase chain reaction-restriction fragment length polymorphism (PCR-RFLP) with the genotyping success rates of 95%–100% and repeatability rates of 98%–100%. Directly DNA sequencing was used to further confirm the genotypes for each SNP for 5 percent of the cases and controls with the concordance rates between RFLP and DNA-sequencing of 98–100%.

### Statistical analysis

The clinical and laboratory data were expressed as mean ± SD or percentage. Genotype distributions were tested at each polymorphic locus for departure from Hardy-Weinberg equilibrium. linkage disequilibrium (LD) and haplotype analysis were carried out for all samples using Haploview 4.2 (Haplotypes are estimated using an accelerated EM algorithm).

Allele frequencies were determined by gene counting. Statistical analyses of association of SNPs with the risk of CAD were performed using the SPSS statistical package (SPSS version 14.0, USA). Qualitative variables were compared using χ^2^ test, and quantitative variables were compared using independent samples t test or Mann-Whitney U test. The associations between CAD and genotypes were analyzed by multiple logistic regression analysis with adjustment for potential confounder including age, gender, BMI and smoking status. As a descriptive measure of association between genotypes and outcomes, p<0.05 is considered to be statistically significant and odds ratios (ORs) were calculated along with 95% confidence intervals (CIs).

## Results

In the study population, the CAD-positive group had more men and smokers compared to CAD-negative group, while no other significant difference in the phenotypic characteristics was found (the values of fasting plasma glucose after intervention) (Table 1).

**Table 1:** The phenotypic characteristics of the study population.

	CAD-positive	CAD-negative	*P*
**N**	425	258	
**Gender (M, %)**	62.1	44.2	<0.001
**Age (y)**	63.2±9.1	62.0±10.0	0.06
**BMI (kg/m2)**	26.1±3.5	25.8±3.7	0.39
**T2DM duration (y)**	8.44±7.1	8.51±7.0	0.76
**FPG (mmol/L)**	7.2±2.6	6.9±2.1	0.35
**Dislipidemia History (positive, %)**	78.7	74.0	0.24
**Hypertension history (positive, %)**	78.3	73.1	0.18
**Smoking history (positive, %)**	50.2	29.3	<0.001

Data are presented in mean ±s.d. BMI: body mass index; FPG: fasting plasma glucose; CAD: coronary artery disease; T2DM: type 2 diabetes. The fasting plasma glucose values were after intervention. Independent t test was used to evaluate age and BMI; Mann-Whitney U test was used to compare the difference of T2DM duration and FBG between the two groups.

### Characteristics and LD structure of the PCSK1 gene

Information of the 5 picked SNPs and p value of Hardy-Weinberg equilibrium test were summarized in Table 2. Genotype distributions among the study population were in agreement with Hardy-Weinberg equilibrium at all 5 loci.

**Table 2:** PCSK1 SNPs Information.

Name	Position	Function	Major/minor allele	minor allele frequency(%)	HW *P* value
**rs6230**	95794603	Intron	T/C	28.6	0.21
**rs6233**	95758868	Exon	T/C	25.4	0.82
**rs6234**	95754730	Exon	C/G	43.9	0.52
**rs156019**	95773119	Intron	T/A	33.2	0.80
**rs3811951**	95788233	Intron	A/G	34.0	0.85

Position information was got from Hapmap. HW *P* value: *P* values of the Chi square test for Hardy–Weinberg equilibrium.

### Polymorphism distributions and association study

We analyzed the distribution of the 5 chosen SNPs in PCSK1 gene in the study population (Table 3). The frequencies of the minor allele G at rs3811951 were significantly different in cases and controls (30.7% vs. 37.2%), the carriers of allele G had a decreased risk for CAD (OR = 0.75, 95%CI = 0.59–0.94, *p* = 0.01). Andrs156019 had a tendency to be associated with CAD risk (*p* = 0.09). The other 3 SNPs had similar allele frequencies between two groups.

**Table 3:** Association of allele frequencies at 5 SNPs with CAD in type 2 diabetic patients.

SNPs (Minor allele)	CAD-positive	CAD-negative	OR	95%CI	*P*
**rs6230(C)**	29.5	26.9	1.14	0.89–1.45	0.30
**rs6233(C)**	26.1	27.8	1.11	0.86–1.42	0.44
**rs6234(G)**	32.6	36.2	0.85	0.67–1.07	0.17
**rs156019(A)**	45.6	40.9	1.21	0.97–1.52	0.09
**rs3811951(G)**	30.7	37.2	**0.75**	**0.59–0.94**	**0.01**

CAD: coronary artery disease; OR: odds ratio; CI: confidence interval. Chi square was used to evaluate OR, 95%CI and *P* value.

Correlations of the SNPs with CAD susceptibility were further explored by the modes of inheritance. In additive mode, the carriers of AT at rs156019 were more susceptible to CAD than the carriers of TT (OR = 1.69, 95%CI = 1.19–2.40, p = 0.003; OR = 1.92, 95%CI = 1.23–3.00, p = 0.04 after adjustment). At the locus rs3811951, GG vs. AG (OR = 0.54, 95%CI = 0.32–0.89, p = 0.02; OR = 0.47, 95%CI = 0.24–0.90, p = 0.02 after adjustment) and GG vs. AA (OR = 0.47, 95%CI = 0.28–0.79, p = 0.004; OR = 0.64, 95%CI = 0.46–0.89, p = 0.008 after adjustment) showed statistically differences, both of which implied that the minor allele G was a protective factor for CAD (Table 4). In recessive mode, the carriers of GG at rs3811951 had a lower risk of CAD (OR = 0.50, 95%CI = 0.31–0.82, *p* = 0.005) compared to carriers of AG or AA, even after adjusted for the other known CAD risk factors, e.g. gender, age, BMI and smoking histories (OR = 0.430, 95%CI = 0.24–0.77, *p* = 0.004 after adjustment).Also in recessive mode, The minor allele G at rs6234 had a tendency to reduce CAD susceptibility of T2DM patients (OR = 0.66, 95%CI = 0.41–1.07, p = 0.09; OR = 0.59, 95%CI = 0.33–1.03, *p* = 0.06 after adjustment) (Table 5). This tendency is more notable in women (OR = 0.416, 95%CI = 0.18–0.95, p = 0.036 after adjustment). In dominant inheritance mode, the SNP rs156019 was associated with CAD risk. The carriers of minor allele A had an increased susceptibility to CAD (OR = 1.575, 95%CI = 1.13–2.19, p = 0.007; OR = 1.659, 95%CI = 1.101–2.502, p = 0.016 after adjustment) (Table 6). No significant difference was found in rs6230 or rs6233 between the cases and controls.

**Table 4:** The association of SNPs with CAD risk in additive inheritance mode.

SNPs	CAD-positive (n = 425)	CAD-negative (n = 258)		P	OR	95%CI	P_a_	OR_a_	95%CI_a_
**rs6230**									
**CC**	36(8.5)	13(5.0)	**CC: CT**	0.10	1.75	0.89–3.44	0.30	1.54	0.68–3.48
**CT**	179(42.1)	113(43.8)	**CC: TT**	0.10	1.74	0.89–3.40	0.39	1.19	0.80–1.77
**TT**	210(49.4)	132(51.2)	**CT: TT**	0.98	1.00	0.73–1.38	0.69	1.09	0.72–1.63
**rs6233**									
**CC**	28(6.6)	15(5.8)	**CC: CT**	0.85	1.07	0.54–2.10	0.52	1.37	0.52–3.59
**CT**	166(39.0)	95(36.8)	**CC: TT**	0.60	1.20	0.62–2.31	0.60	1.14	0.71–1.82
**TT**	231(56.4)	148(57.4)	**CT: TT**	0.50	1.12	0.81–1.55	0.99	1.00	0.67–1.49
**rs6234**									
**GG**	40(9.4)	35(13.6)	**GG: GC**	0.134	0.68	0.41–1.13	0.09	0.59	0.32–1.08
**GC**	197(46.4)	117(45.3)	**GG: CC**	0.09	0.64	0.39–1.08	0.06	0.74	0.54–1.01
**CC**	188(44.2)	106(41.1)	**GC: CC**	0.76	0.95	0.68–1.32	0.63	0.90	0.59–1.37
**rs156019**									
**AA**	82(19.3)	51(19.8)	**AA: AT**	0.25	1.28	0.84–1.94	0.11	1.54	0.91–2.58
**AT**	224(52.7)	109(42.2)	**AA: TT**	0.21	1.32	0.85–2.06	0.553	1.18	0.68–2.04
**TT**	119(28.0)	98(38.0)	**AT: TT**	**0.003**	**1.69**	**1.19–2.40**	**0.04**	**1.92**	**1.23–3.00**
**rs3811951**									
**GG**	35(8.2)	39(15.1)	**GG: AG**	**0.02**	**0.54**	**0.32–0.89**	**0.02**	**0.47**	**0.24–0.90**
**GA**	191(45.0)	114(44.2)	**GG: AA**	**0.004**	**0.47**	**0.28–0.79**	**0.008**	**0.64**	**0.46–0.89**
**AA**	199(46.8)	105(40.7)	**AG: AA**	0.47	0.88	0.64–1.23	0.65	0.90	0.57–1.41

CAD: coronary artery disease; OR: odds ratio; CI: confidence interval. OR_a_, CI_a_ and *P*_a_ reperesent OR, CI, p after adjustment for gender, age, BMI and smoking history. Chi square test was used to evaluate OR, 95%CI and *P* value. multiple logistic regression analysis was used to analyze OR_a_, 95%CI_a_ and *P*_a_.

**Table 5:** The association of SNPs with CAD risk in recessive inheritance mode.

SNPs	CAD-positive (n = 425)	CAD-negative (n = 258)	P	OR	95%CI	P_a_	OR_a_	95%CI_a_
**rs6230**			0.09	1.74	0.91–3.36	0.35	1.45	0.67–3.16
**CC**	36(8.5)	13(5.0)						
**TX**	389(91.5)	245(95.0)						
**rs6233**			0.69	1.14	0.60–2.18	0.54	1.33	0.53–3.38
**CC**	28(6.6)	15(5.8)						
**TX**	397(93.4)	243(94.2)						
**rs6234**			0.09	0.66	0.41–1.07	0.06	0.59	0.33–1.03
**GG**	40(9.4)	35(13.6)						
**CX**	385(90.6)	223(86.4)						
**rs156019**			0.88	0.97	0.66–1.43	0.49	0.90	0.53–1.36
**AA**	82(19.3)	51(19.8)						
**TX**	343(80.7)	207(80.2)						
**rs3811951**			**0.005**	**0.50**	**0.31–0.82**	**0.004**	**0.43**	**0.24–0.77**
**GG**	35(8.2)	39(15.1)						
**AX**	390(91.8)	219(84.9)						

CAD: coronary artery disease; OR: odds ratio; CI: confidence interval. OR_a_, CI_a_ and *P*_a_ reperesent OR, CI, *P* after adjustment for gender, age, BMI and smoking history. OR, 95%CI and *P* value were compared using Chi square test. OR_a_, 95%CI_a_ and *P*_a_ were analyzed by multiple logistic regression analysis.

**Table 6:** The association of SNPs with CAD risk in dorminant inheritance mode.

SNPs	CAD-positive (n = 425)		CAD-negative (n = 258)	P	OR	95%CI	P_a_	OR_a_	95%CI_a_
**rs6230**				0.66	1.07	0.79–1.46	0.99	1.00	0.68–1.47
**CX**	215(50.6)		126(48.8)						
**TT**	210(49.4)		132(51.2)						
**rs6233**				0.44	1.13	0.83–1.54	0.99	1.02	0.68–1.47
**CX**	194(54.4)		110(57.4)						
**TT**	231(45.6)		148(42.6)						
**rs6234**				0.42	1.17	0.83–1.56	0.30	1.24	0.83–1.84
**GX**	237(55.8)		152(57.9)						
**CC**	188(44.2)		106(42.1)						
**rs156019**				**0.01**	**1.54**	**1.13–2.19**	**0.01**	**1.76**	**1.18–2.64**
**AX**	306(72.0)		160(62.0)						
**TT**	119(28.0)	98(38.0)							
**rs3811951**				0.12	0.78	0.57–1.07	0.09	0.71	0.48–1.05
**GX**	226(53.2)		153(59.3)						
**AA**	199(46.8)		105(40.7)						

CAD: coronary artery disease; OR: odds ratio; CI: confidence interval. OR_a_, CI_a_ and *P*_a_ reperesent the OR, CI and p values after adjustment from gender, age, BMI and smoking history, respectively. Chi square test was used to evaluate the 95% CI and P value. Multiple logistic regression analysis was used to evaluate the OR_a_, 95%CI and *P* values.

### Haplotype analysis

Haplotypes were constructed using rs6234, rs6233, rs156019 and rs3811951 depending on the physical position and the value of D′ (D′>0.5) between every two SNPs in one block. Common haplotypes constructed by the 4 SNPs were GTTA (30.5%), CTAG (23.9%), GCAA (16.0%) and GTAA (9.7%) (Table 7). And the haplotype CTAG shows a significant association with CAD, the carriers of CTAG had a lower risk of CAD compared to non-carriers (OR = 0.686, 95%CI = 0.54–0.88, P = 0.02).

**Table 7:** Association of the frequencies of common haplotypes with CAD.

haplotype	rs6234	rs6233	rs156019	rs3811951	CAD-positive (%)	CAD-negative (%)	*P*
1	G	T	T	A	31.0	29.7	0.61
2	C	T	A	G	21.1	28.5	0.02
3	G	C	A	A	16.1	15.8	0.86
4	G	T	A	A	10.3	8.8	0.37

CAD: coronary artery disease. Chi square test was used for statistical analysis.

## Discussion

In this study, we have demonstrated that three genetic variants of PCSK1, namely rs3811951 (A>G), rs156019 (A>T), and rs6234 (C>G) were associated with the risk of CAD in T2DM in Chinese population, with the association being stronger for rs3811951 and rs156019.

In support of our findings, loss-of-function mutations in PCSK1 gene caused proinsulin accumulation and insulin deficiency [7]–[9], and plasma glucagon-like peptide-1 level increased after transplantation of PCSK- encoded PC1/3- expressing pancreatic α-cells in mice [10]. Both proinsulin, insulin and GLP-1 is closely related to T2DM and CAD. While insulin can protect heart through PI3K–Akt–eNOS–NO pathway which can increase the NO production and is independent of glucose presence [11], the increased concentration of proinsulin in serum is an independent predictor of CAD [12], [13]. At the same time, it has been demonstrated that GLP-1 could inhibit the formation of atheromatous plaques by its upregulation of NO production in endothelium, vascular smooth muscle cells, monocytes and macrophages [5].

Several studies on association of PCSK1 genetic vairants with metabolic traits had been done, but no study on the association of genetic variability in PCSK1 gene with CAD risk in the population with type 2 diabetes has been reported. We identified a suggestive association of genetic variants at rs3811951 with CAD susceptibility. Chang and Chiu et al. found rs3811951 was associated with fasting insulin, triglycerides, and high-density lipoprotein cholesterol (P = 0.05, 0.003, 0.001, 0.04, and 0.04, respectively) [14], which may indicate the possible association of PCSK1 with risk of CAD. In Chang's study [14], rs155971 v, which is in strong LD with rs156019 (r^2^ = 1 in hHapMap CHB database), was found associated with obesity, while in our study carriers of A allele of rs156019 had a higher CAD risk. Among all the studies on SNPs of PCSK1 gene, intense attentions have been paid to PCSK1 nonsynonymous variants rs6232, rs6234 and rs6235, which are located in exons. A study published in 2011 showed that these 3 SNPs may affect the biosynthesis and activity of PC1/3 coded by PCSK1 gene [15]. As rs6232 was not found in Chinese population and rs6234 was in strong LD with rs6235 (r^2^ = 1 in HapMap CHB database ), we genotyped rs6234 (encoding Q665E) only and found that carriers of minor allele G at rs6234 had a tendency to be less susceptible to CAD in recessive inheritance mode. A study on association of PCSK1 rs6234 with obesity and related traits in Chinese population found that allele G was associated with increased beta-cell function estimated by HOMA-S and HOMA-B [16]. Case-control association studies in Europe also indicated that rs6234/rs6235 was associated with fasting insulin and HOMA-IR [17]–[19].

It is known that nonsynonymous substitutions such as rs6234 can change the amino acid coding and participate in pathogenesis. However, the mechanisms by which SNP rs156019 and rs3811951, both located in the intronic regions, affect the CAD risk were not known yet. We postulate that the related SNPs may modify the expression levels of mRNA and even coding-proteins, which leads to decreased activity of PC1/3. But it's more likely that the SNPs are only genetic markers in strong LD with other pathogenic variants.

However, the following limitations should be acknowledged in the study. The sample size was small and the clinical features weren't perfectly matched between the case and control groups, which might affect the results. This hospital is a first class tertiary hospital and most people came here for a treatment of their advanced illnesses, the number of CAD-negative patients in the control group is relatively small and may not be similar to the community-based case-control study. Further functional studies should be considered on the genetic variants in PCSK1 gene. It needs to be confirmed in a prospective study before PCSK1 polymorphisms are used to predict the risk of CAD in type 2 diabetes in the Chinese population.

## Conclusions

We found modest evidence for association of the PCSK1 variants rs6234, rs156019 and rs3811951 with CAD risk in Chinese Han population with T2DM, which may contribute to the illustration of the common genetic pathogenesis of CAD and T2DM and provide new targets to screen out the individuals with T2DM at high risk of CAD.
